# A Dual Cluster-Head Energy-Efficient Routing Algorithm Based on Canopy Optimization and K-Means for WSN

**DOI:** 10.3390/s22249731

**Published:** 2022-12-12

**Authors:** Mei Wu, Zhengliang Li, Jing Chen, Qiusha Min, Tao Lu

**Affiliations:** 1 School of Computer Science and Engineering, Wuhan Institute of Technology, Wuhan 430205, China; 2 School of Mathematics and Physics, Wuhan Institute of Technology, Wuhan 430205, China; 3 School of Educational Information Technology, Central China Normal University, Wuhan 430079, China

**Keywords:** wireless sensor networks, network lifetime, canopy algorithm, K-means algorithm, energy-efficient

## Abstract

Wireless sensor networks (WSN) are widely used in various applications, such as environmental monitoring, healthcare, event detection, agriculture, disaster management, and so on. Due to their small size, sensors are limited power sources and are often deployed in special environments where frequent battery replacement is not feasible. Therefore, it is important to reduce the energy consumption of sensors and extend the network lifetime. An effective way to achieve this is clustering. This paper proposes a dual cluster-head energy-efficient algorithm (DCK-LEACH), which is based on K-means and Canopy optimization. Considering that the K-means algorithm is sensitive to the location of the initial clustering centers, this paper uses both the dynamic Canopy algorithm and K-means algorithm for clustering. For cluster-head election, this algorithm uses a hierarchy to minimize the cluster-head burden and balance the network load. The primary cluster-head is selected by two objectives: the node’s residual energy and the distance from the node to the clustering center. The vice cluster-head is selected by the residual energy of the node, and the distance from the nodes to the base station. Simulator results show that DCK-LEACH significantly prolongs the energy-critical node lifetime and the network lifetime compared with existing protocols.

## 1. Introduction

A wireless sensor network consists of a large number of spatially distributed microelectronic devices [1,2,3]. These nodes sense elements of their environments and communicate among their peers, or directly transmit data to the base station. During the process of communication, nodes consistently consume energy most of the time. However, a node’s battery energy is limited, and nodes are usually deployed in open, complex areas, where batteries are not easily replaced or recharged [4]. Once a node’s energy is exhausted, it stops transmitting data, which is equivalent to the “death” of the node. When more than one node “dies”, the network is no longer connected, eventually resulting in the entire wireless sensor network becoming “paralyzed”. Therefore, the key point lies in reducing the energy consumption of nodes and extending the lifetime of the network in terms of routing protocols in wireless sensor networks.

In wireless sensor networks, energy-efficient routing protocols aim to use the least amount of energy to transmit data from the sender to the receiver.

In clustering routing algorithms, a sensor network area is divided into clusters, and data is transmitted to the base through these clusters. Clustering is designed to extend the network lifetime by balancing energy consumption [5]. 

The low-energy adaptive hierarchical clustering protocol (LEACH) [6] is a popular clustering routing protocol, which randomly selects cluster-heads. When a node with low energy is selected as a cluster-head, that node will quickly consume more energy and die earlier than normal nodes, which accelerates the “death” of the entire network. Some researchers have tried to tackle this problem. In [7], the authors proposed a protocol based on LEACH. In the cluster-head selection, it considers the maximum residual energy of nodes, so cluster-heads can work for a long time compared with that of LEACH. In [8], the authors proposed the BRE-LEACH protocol that aims to extend the network lifetime by optimizing energy consumption. The selection of cluster-heads is based on three main factors: the residual energy of nodes, the distance to the base station (BS) from the nodes, and the number of hops. Then, the optimal path is formed to forward the data to the BS in the network. 

However, the above methods only aim to select the appropriate cluster-heads, and then to cluster nodes according to their distances from the cluster-head. They did not consider that the maximum or the minimum cluster will be generated in the network, which may lead to uneven energy consumption between clusters because the energy consumption of larger clusters is greater than that of smaller clusters. 

Therefore, in this paper, we firstly consider the cluster size in clustering, and make the clusters relatively equal in size using our algorithm, to balance the energy consumption of the network. Then we consider a dual cluster-head approach to share the workload of the original single cluster-head. The dual cluster-head approach further extends the lifetime of the cluster-head and reduces the energy waste caused by frequent repetitive clustering.

Nowadays, one of the popular clustering strategies is the K-means algorithm. The K-means (KOCED) protocol for optimal clustering of wireless sensor networks was proposed in [9]. It uses the K-means algorithm to cluster nodes, and the clusters formed are more uniform compared with the clusters of the LEACH protocol. This may greatly reduce the energy consumption of cluster-heads.

However, when constructing clusters using the K-means algorithm, the value of K needs to be predefined and may not always be reasonable for the network. In addition, since the K-means algorithm is very sensitive to the location of the initial cluster centers, its initial K-cluster centroids are randomly elected at the time, which may cause it to fall into local optimization [10,11] and subsequently form maximum or minimum clusters. This leads to load imbalance between clusters. 

The Canopy algorithm is a “coarse” clustering algorithm, and its clustering speed is fast. Unlike traditional clustering algorithms, the number of clusters (i.e., k-value) does not need to be specified in advance for Canopy clustering [12]. When the algorithm combines the Canopy algorithm and K-means algorithm, it will overcome the shortcoming of the K-means algorithm that the k-value needs to be specified artificially, and improve the algorithm’s clustering accuracy. It can effectively balance the energy load of the network.

This paper proposes an energy-efficient routing algorithm (DCK-LEACH), which is based on dual cluster-head (CH), Canopy optimization, and K-means algorithm. The algorithm imports a hybrid Canopy optimization and K-means algorithm for creating clusters and dynamically selecting the primary and vice cluster-heads by self-adaptive means. It effectively reduces the burden of cluster-heads and prevents them from dying too quickly. Thus, the lifetime of the network is extended.

The main contributions of this paper are summarized as follows.

(1) The DCK-LEACH algorithm is a concentration strategy to construct clusters in the network, by using a mixture of Canopy optimization and the K-means algorithm. It avoids the problem of specifying the value of K. Compared with using one of them, DCK-LEACH combines these two algorithms and is faster in terms of clustering speed, and its accuracy is higher. It also distributes the energy more evenly between clusters.
(2) DCK-LEACH uses a hierarchical structure consisting of a primary CH and a vice CH in each cluster to reduce the communication load on cluster-heads. Dual cluster-heads together share the workload a single cluster-head would previously have taken on; this makes the energy balanced within the cluster and reduces the communication load of the cluster-head.
(3) A cluster-head selection algorithm based on the fitness function is proposed, which dynamically self-adjusts the weight coefficients of the fitness function according to the change of residual energy in the network. Here, (1) since the primary cluster-head is responsible for communicating with nodes in the cluster, it is better to place the primary cluster-heads in the center of the cluster, and for them to have a higher residual energy. As a result, the energy consumed by the primary CH is uniform and its survival time is extended in the network. (2) The vice CH is responsible for forwarding data to the base station, and is best placed close to the base station so that it consumes less energy.
(4) We propose a concept of cluster-head selection, which is similar to the LEACH algorithm, to control the frequency of re-clustering operation and thus avoid energy waste, because not all operations of clustering and cluster-heads replacement need to be performed in every round. In each round, when the base station performs clustering and cluster-heads selection operations, all nodes must send their own information (i.e., location and remaining energy) to the base station, which will consume a lot of energy.
(5) Using simulation software, the proposed DCK-LEACH algorithm is compared with four conventional algorithms such as LEACH, RCH-LEACH, IEECHS-WSN, and K-LEACH.

The remainder of this paper is organized as follows. The related works are detailed in Section 2. Section 3 presents the system model of the proposed algorithm. Section 4 describes the proposed algorithm in detail. In Section 5, the performance of the proposed method is investigated. In Section 6, the proposed method is discussed. Finally, the paper is concluded in Section 7.

## 2. Related Works

To reduce the energy consumption of nodes and prolong the network lifetime, a large number of hierarchical routing protocols have been proposed by clustering or using topology control to keep the transmission distance low [13].

One of the most classical clustering routing protocols is LEACH, proposed by Heinzelman et al. [6]. In the LEACH protocol, the random selection of cluster-heads [14] it may lead to the rapid death of cluster-heads by electing the lower energy node as a cluster-head. It divided the wireless sensor network into several clusters, and one cluster-head was randomly selected in each cluster. Each node had equal opportunity to be selected as a cluster-head.

A hybrid low energy adaptive clustering hierarchy protocol (H-LEACH) has been proposed [15], which added the average residual energy of all nodes in a network to the threshold formula, and the node was defined as a cluster-head when its residual energy was greater than this threshold. Selecting the nodes with higher residual energy as a cluster-head avoided the lower energy nodes becoming cluster-heads and dying too fast.

In [16], a residual energy-based cluster-head selection algorithm (RCH-LEACH) was proposed. It calculates the energy threshold for selecting cluster-heads based on the 5–10% ratio range of cluster-heads. When the residual energy of nodes is greater than this threshold, nodes are selected as cluster-heads, which makes the energy between clusters more balanced in the network.

An improved integrated LEACH protocol was proposed in [17]. It combines the A* search algorithm with the integrated LEACH protocol to form a tree of sensor nodes, then selects nodes as the cluster-heads in the shortest path. This minimizes the number of deaths of sensor nodes and saves the energy of sensor nodes.

In [18], a hybrid routing algorithm (HRA-NP) was proposed based on a plain Bayesian and improved particle swarm optimization algorithm (PSO). Firstly, cluster-heads (CH) are selected based on conditional probabilities, which are estimated by a plain Bayesian classifier. Then, the multi-hop routing algorithm is applied to the data delivery of the cluster-heads. It finds the optimal path from each CH to the BS using the PSO algorithm.

In [19], to reduce the energy consumption of the network, the U-LEACH (Universal-Low Energy Adaptive Cluster Hierarchy) algorithm was proposed, which forms a cluster-head chain connected to the base station. 

In [20], we proposed a hybrid optimization routing algorithm based on improved GA and ACO in heterogeneous sensor networks. The combination of the GA algorithm and ACO algorithm is used to select better routes. The network lifetime is effectively extended.

To solve the problem of the premature death of cluster-heads due to very fast energy consumption and the unbalanced energy consumption of nodes, a double cluster-head routing algorithm was proposed in [21]. The primary cluster-heads and member nodes were identified through the LEACH protocol, and the vice cluster-heads were selected among the cluster members within the same cluster based on the residual energy of the nodes. 

In [22], an improved energy-efficient dual cluster-head selection protocol (IEECHS-WSN) was proposed. In cluster-head election, two cluster-heads were selected in the same cluster. The election of the first cluster-head is based on a threshold approach similar to that of LEACH, and it primarily transfers data from the cluster to the base station. The second cluster-head received data from other nodes in the cluster, then fused and forwarded it to the first cluster-head. The method selected the node with the closest distance to the cluster’s centroid as the second cluster-head. Therefore, the energy of the cluster-heads was not consumed too quickly, which effectively extended the network lifetime and reduced the energy consumption in IoT applications. 

On the other hand, a K-means clustering-based K-LEACH algorithm was proposed to extend the network lifetime by balancing the energy consumption of nodes [23]. In K-LEACH, the K-means method was used for uniform clustering during network initialization, and cluster-heads were selected based on the maximum residual energy of nodes and the nearest distance between the nodes and the cluster centers. As a result, the nodes with more uniform membership and larger residual energy were more likely to be selected as cluster-heads. 

Generally, the K-means clustering algorithm uses the distance formula to calculate whether two nodes belong to the same cluster. The nodes within the threshold of distances join in the same cluster, and the nodes out of this scope are divided into other clusters. Therefore, this method makes the distance between the cluster-head and the member nodes of the cluster in a reasonable range.

In [24], a Q-learning routing algorithm based on the K-means algorithm was proposed for optimal clustering and energy load balancing in wireless sensor networks. Firstly, the Q-learning algorithm is used for training to obtain the optimal initial parameters of the K-means algorithm, which enables the K-means algorithm to obtain better clustering results. Secondly, it also considers the area of clusters and the number of sensors in the clusters, where nodes with large residual energy and a small distance to the receiving node are selected as cluster-heads. This effectively extends the network lifetime. However, it is not ready-to-use and cannot be deployed in various scenarios because the Q-learning algorithm needs be trained in advance.

In [25], an energy aware distance-based cluster-head selection and routing (EADCR) protocol was proposed, which used the fuzzy C-mean (FCM) algorithm, residual energy of the nodes, their relative Euclidean distances from the base station (BS), and cluster centroid to extend the network lifetime. It used the shortest path algorithm among nodes to reduce the energy consumption of cluster-heads via multi-hop routing. 

In [26], a new adaptive and hybrid clustering scheme was proposed that used the Euclidean distance, the fuzzy C-mean (FCM) algorithm, the position of the BS, and the residual energy of the nodes to cluster the nodes, and therefore to minimize the energy consumption of nodes. 

Clustering was only performed in a few rounds, which significantly reduced the energy consumption. The scheme used an energy-efficient fitness function to select cluster-heads in the network. This effectively extended the network lifetime.

The above research were mainly focused on routing algorithms on static base stations (sinks), which have unlimited energy and powerful computing power. It aimed to minimize energy loss and extend the network lifetime where the sensor’s energy cannot be replenished. 

On the other hand, some researchers work on mobile sinks, where the static base station is replaced by an unmanned aerial vehicle (i.e., drones) [27,28], and tackle the Traveling Salesman Problem (TSP) between the sink and cluster-heads. 

In [27,28], the authors proposed two sustainable and quality-aware data collection algorithms (QEADCS, GAMEDFS) in the UAV-assisted manner to reduce energy consumption in robotic wireless sensor networks. They consider the problem under the constraint on the limited-capacity battery of UAV, so the UAV would not access all CH robots. In each cluster, a cluster-head (CH) robot assigns a collaborative task to cluster member (CM) robots and collects data from the CMs, while an unmanned aerial vehicle (UAV) collects data from the CHs robot by accessing a subset of them. The total cost in routing is minimized by optimizing the UAV path planning to minimize its data quality and energy consumption. It used the shortest path TSP algorithm among UAV and CH nodes to reduce the energy consumption of cluster-heads via multi-hop routing.

## 3. System Model of DCK-LEACH

This section describes the system model of DCK-LEACH in detail. Firstly, the network model and the energy consumption model are introduced, respectively. Then, some definitions and assumptions are presented.

### 3.1. Network Model

Our algorithm is applicable to static networks where there are one base station and n static nodes. We assume that a wireless sensor network is a two-dimensional plane. There is a fixed base station for receiving data from nodes, and n randomly distributed sensor nodes for collecting information. 

We make the following assumptions for simulating the network.

(1) The sensor nodes are randomly distributed in a two-dimensional plane, and they are static, thus will not move once they are deployed.
(2) In the monitoring range, all sensor nodes are homogeneous, which have the same initial energy, data processing, and communication capabilities.
(3) Sensor nodes have the ability to access to their own location in the network, their own residual energy, and the ability to bring cluster-heads to fuse redundant data.
(4) The base station has an unlimited power supply and computing power, and each node can communicate directly with this base station.

**Figure 1:**
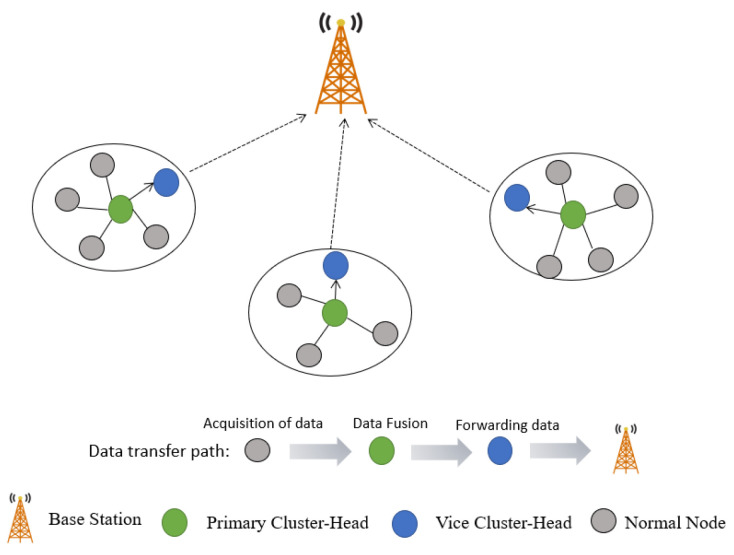
Network topology of the proposed algorithm.

Figure 1 shows the network topology of the proposed algorithm. There are three phases in the algorithm: the cluster establishment phase, cluster-head selection phase, and stable operation phase.

In the cluster establishment phase, rough clustering is preformed in terms of the Canopy algorithm, and then precise clustering is preformed using the K-means algorithm. We finally obtained K clusters and the cluster IDs with the nodes they belonged to in a network. The cluster-head selection phase includes two elections: the primary cluster-head, and its vice cluster-head in the same cluster. The election of the primary cluster-head is decided by two factors: the residual energy of the node, and the distance from the node to the cluster center. The vice cluster-head is selected based on two factors: the residual energy of the node, and its distance from the base station.

In the stable operation phase, data which is collected from the inter-cluster normal nodes are forwarded to the primary cluster-heads, and then to the vice cluster-heads, and finally are forwarded to the base station. The intra-cluster data are forwarded in a hierarchy (see Figure 1), where normal nodes collect data, and forward them to the primary cluster-head in the cluster where the node belongs. The primary cluster-head aggregates data and transmits it to the vice cluster-head, and the latter receives the data and sends it to the base station.

### 3.2. Energy Consumption Model

In this paper, the network energy model uses the same radio energy model as the one defined in LEACH [29,30]. When the communication distance between two communicated nodes is greater than the threshold $d_{0}$, the communication energy model uses the multipath fading channel model. When the communication distance between two communicated nodes is less than the threshold $d_{0}$, the communication energy model uses the free-space channel model, where the threshold $d_{0}$ is shown in Equation (1).

(1) $$d_{0}=\sqrt{\frac{\varepsilon_{fs}}{\varepsilon_{mp}}}$$

where $\varepsilon_{fs}$ and $\varepsilon_{mp}$ are denoted as the amplification factor parameters of the free-space channel model and the multi-path fading model, respectively.

The energy consumption $E_{tx}$ of a node for sending *m* bits of data to a node at distance *d* is as follows:

(2) $$E_{tx}\left(m,d\right)=\{\begin{array}{c} mE_{elec}+m\varepsilon_{fs}d^{2},d<d_{0}\\ mE_{elec}+m\varepsilon_{mp}d^{4},d\geq d_{0} \end{array}$$

where $E_{elec}$ denotes the energy consumption required by a node to send or receive a packet, and d denotes the distance between the transmitting node and the receiving node.

The energy consumed by a sensor node for receiving data is independent of the distance between the transmitting and receiving nodes. The energy consumed by the node to receive m bits of data is as follows:

(3) $$E_{rx}=mE_{elec}$$

where $E_{rx}$ indicates the energy consumed by the node to accept the packet.

## 4. Methods

In this paper, an energy-balancing algorithm DCK-LEACH is proposed to extend the network lifetime by balancing the load of the whole network.

### 4.1. Cluster Establishment Phase

How are the sensor nodes clustered? Nodes are assigned to different clusters according to the similarity between the nodes. In this paper, we use the K-means algorithm based on Canopy to cluster all nodes in WSN.

#### 4.1.1. Coarsely Clustering All Nodes to Obtain K Initial Clustering Centers

In this paper, the Canopy algorithm is used to pre-cluster nodes, and to quickly obtain the k-value and *k* initial cluster centroids. Then, the output is used as the input of the initial parameters for the K-means algorithm to cluster nodes in WSN. 

The process of the Canopy algorithm in DCK-LEACH for optimizing the initial clustering centers is as follows.

Step 1: Randomly choose a node in the set N, N = {Node (1), Node (2), …, Node (n)}, denoted as P1 and delete P1 in N. Mark P1 as a cluster center point of Canopy, and mark it as Canopy (1). Save the node position information into the set C = {Canopy (1), Canopy (2), … Canopy (*i*)}.

Step 2: Set the value of thresholds T1 and T2 (T1 = 50, T2 = 0.5 T1), traverse the set N, and calculate the distances of the nodes in N to the nodes in the set C using Equation (4), respectively. If the distance of node *i* to a Canopy (*i*) in the set C is less than T1, the cluster ID of the node *i* is named *i*. If the distance of node j to a Canopy (*i*) in the set C is less than T2, it means that node j is very close to Canopy (*i*), and the node j is removed from N to avoid repeatedly joining it to other clusters.

Step 3: if the distance of the node j to all existing Canopy centroids is greater than T1, we add node j to the set C as a new Canopy centroid.

Step 4: Iterate through the residual nodes in N, continue with Step 2 and Step 3 until N is empty, and get the set C of all Canopy centroids and the number of clustering centers (K). Use the above result as the input of the initial parameter of the K-means algorithm for further clustering.

#### 4.1.2. Clustering the Network

In this paper, we use Euclidean distance to calculate the distance function. The clustering steps based on K-means clustering algorithm are as follows.

Step 1: the base station randomly selects K nodes among N nodes as the initial clustering centers.

Step 2: for other nodes, calculate the distances between them and the K initial clustering centers (dist) in step 1 according to the formula in Equation (4) [31].

(4) $$dist\left(t_{i},t_{j}\right)=\sqrt{{\left(x_{i}-x_{j}\right)}^{2}+{\left(y_{i}-y_{j}\right)}^{2}}$$

where $d\left(t_{i},t_{j}\right)$ denotes the Euclidean distance from node $t_{i}$ to node $t_{j}$, and $x_{i}$ and $y_{i}$ are the horizontal and vertical coordinates of $t_{i}$, respectively. 

Step 3: assign the nodes, which are close to the cluster center, to the nearest cluster where the corresponding cluster center is located.

Step 4: use Equation (5) [32] to calculate the mean value of the new cluster after assignment and update the clustering center.

(5) $$C_{j}=\frac{1}{N_{j}}\underset{i=1}{\overset{N_{j}}{\sum^{\;}}}x_{i}^{\left(j\right)},j=1,2,3\ldots ,K$$

Step 5: repeat Step 2, Step 3, and Step 4 until the number of iterations exceeds the predefined value, then the process of clustering is ended.

When the network is initialized, the base station assigns unique IDs to all nodes to identify their respective identities. The base station uses the K-means algorithm to cluster based on the node’s location, repeatedly iterates to the maximum time which is given prior to the construction of the network, and finally broadcasts the results of clustering to all nodes in WSN.

### 4.2. Cluster-Head Election Phase

This paper develops a dual cluster-heads method combining the primary and vice cluster-heads, to reduce the communication load of cluster-heads, and extend the network lifetime. The primary cluster-head is selected based on a combination of the node’s residual energy and the distance from the node to the clustering center. The vice cluster-head is selected based on two objectives: the node’s residual energy, and the distance from the node to the base station.

#### 4.2.1. Elect the Primary Cluster-Heads in DCK-LEACH

It is necessary to elect the optimal node as cluster-heads to extend the network lifetime. Because cluster-heads spend more energy compared with that of other nodes in the cluster when they receive, fuse, and forward data within the cluster or among clusters. 

In selecting the primary cluster-heads, the fitness function (*p*) is defined by two factors: the residual energy of the node, and the distance from the node to the clustering center. In addition, the weight probabilities of factors are dynamically adjusted according to the characteristics of the network in real-time. Finally, the node with a larger fitness function (*p*) value is selected as the primary cluster-head in a cluster.

The residual energy of nodes is an important factor in cluster-head election [33,34], and we thus increase the possibility of nodes with high residual energy being elected as the primary cluster-head in DCK-LEACH.

The first objective function ($f_{1}(i))$ [35] for selecting the cluster-head, which is the ratio of the residual energy of a node *i* to the average residual energy of all nodes in the same cluster to which it belongs, is as follows:

(6) $$f_{1}(i)=\frac{E_{res}\left(i\right)}{\frac{1}{n}\underset{j=1}{\overset{n}{\sum }}E_{res}\left(j\right)}$$

where *n* is the number of nodes in the cluster, and $E_{res}\left(i\right)$ is the residual energy of node i. The greater the residual energy of node i, the larger the value of $f_{1}(i)$, and the higher the probability of being elected as the cluster-head for *i*.

After clustering, K clusters and the cluster centers of each cluster were obtained. When the position of the primary cluster-head is closer to the cluster center, the distance from the cluster-head to other member nodes within this cluster is more uniform. 

The second objective function ($f_{2}(i))$ for selecting the cluster-head close to the cluster center is as follows:

(7) $$f_{2}(i)=\frac{\frac{1}{n}\underset{i=1}{\overset{n}{\sum }}d_{tocentre}\left(i\right)}{d_{tocentre}\left(i\right)}$$

where $d_{tocentre}\left(i\right)$ is the distance from node i to the cluster center. The closer node i is to the cluster center, the greater the value of $\;f_{2}$, and the greater the chance of becoming the cluster-head. 

The more reasonable the position of the cluster-head to other member nodes in the cluster is, the lower the sum of the energy consumption of all nodes within this cluster.

Considering the above two factors, nodes with more residual energy and positioned close to the cluster centroids are more likely to become the primary cluster-heads. The fitness function (*p*) for selecting the primary cluster-heads is as follows:

(8) $$P(i)=\alpha f_{1}(i)+\beta f_{2}(i)$$

where *α* and *β* are two weighting coefficients to adjust the weights of these two factors.

In the initial construction of the network, the energy of the nodes is full, and the residual energy of the network is more than half of the initial energy of the network (0.5 * n$E_{0}$). These two factors are equally important when selecting the primary cluster-head. So, the initial setting is α = β = 1/2.

When the residual energy of the network is less than half of the initial energy of the network (0.5 * n$E_{0}$), nodes have low remaining energy, and it is necessary to give priority to the factor of the node’s residual energy compared with the factor of distance from the node to the clustering center. Therefore, the weights of the objective functions $f_{1}(i)$ and $f_{2}(i)$ need to be adjusted, where α and β are as follows:

(9) $$\alpha \left(i\right)=\frac{E_{res}\left(i\right)}{E_{0}}$$

(10) β(i)=1−α(i)

where $E_{0}$ is the initial energy of each node, and $E_{res}\left(i\right)$ is the current residual energy of node i. The greater the residual energy of node i, the larger the value of α(i), and the larger weight value of the objective function $f_{1}(i)$. In short, the node with large residual energy has a higher probability of being selected as the primary cluster-head in this situation.

Algorithm 1 in DCK-LEACH for the primary cluster-heads election is as follows:
**Algorithm 1: The primary cluster-heads election****Input:** all member nodes in K clusters **Output:** the primary cluster-heads in the set C1 ----------------------------------------------------------------------------------------------------------------- (1) **For** each cluster (2)     **For** member nodes within the same cluster (3)          Calculate the residual energy objective function $f_{1}(i)$, and the distance function $f_{2}(i)$ for each node i (4)               **If** the sum of energy of all nodes is greater than half of the initial energy of the network (0.5 * n$E_{0}$) (5)                  Calculate the value of the node adaptation function according to P(i) = 1/2$*f_{1}(i)$ + ½ * $f_{2}(i)$ (6)               **Else** (7)                  Calculate the value of the node adaptation function according to P(i) = $\frac{E_{res}\left(i\right)}{E_{0}}*f_{1}(i)$ + (1 −$\;\frac{E_{res}\left(i\right)}{E_{0}}$) * $f_{2}(i)$ (8)       Find the node with the largest fitness function value and save this node information in the set C1 (9) **End**

For each node *i* in the cluster (*i* = 1… *n*), respectively, calculate the objective functions $f_{1}(i)$ and $f_{2}(i)$, and then select the primary cluster-head based on the maximum fitness function *p*-value among nodes according to the weight of the two objective functions. 

Here, to compare the value of P(i), $f_{1}(i)$, and $f_{2}(i)$ of the member nodes within a cluster, we list the data in one round in scenario 1, which is as follows:

Table 1 shows that the node (ID = 6) has the largest value of the adaptive function *p*. Therefore, the node (ID = 6) becomes the primary cluster-head of this cluster. For the other clusters in the network, the objective function $(f_{1}$,$f_{2})$ and the fitness function value (*p*) are calculated for each node, respectively, and finally the primary cluster-head in each cluster is selected according to the value of the fitness function (*p*).

#### 4.2.2. Elect the Vice Cluster-Heads in DCK-LEACH

Since the cluster-head needs to receive the data from the member nodes in the cluster and communicate with the base station and other cluster-heads, the energy consumption of cluster-heads increases significantly with increasing network scale. 

To address this problem, our solution, which features direct communication with the base station via the vice cluster-head, is proposed to reduce the energy consumption of the primary cluster-head by the vice cluster-head. When the vice cluster-head is elected in the cluster, we assume that the distance between the vice cluster-head and the base station is closer than that of the cluster-head to the base station. Member nodes in a cluster will aggregate their local data and send it to the primary cluster-head in that cluster. The primary cluster-head will transmit the data to the vice cluster-head, and the latter will send it to the base station. This solution reduces the transmission energy consumption of cluster-heads.

In DCK-LEACH, the vice cluster-heads are selected using a weighted probability. This mainly considers two factors: the distance between the node and the base station, and the residual energy of the node. Firstly, it calculates the adaptive degree function (*Q*) in DCK-LEACH by these two factors, and then dynamically adjusts the weight probabilities of the two factors. Finally, the DCK-LEACH algorithm selects the best vice cluster-head with the maximal value of *Q*.

For the first factor, we prefer to choose the node which is as close as possible to the base station among the same cluster, since, as the data transmission distance between nodes increases, the data transmission energy consumption increases exponentially [36]. This will reduce the energy consumption of the vice cluster-head in forwarding packets to the base station.

Algorithm 2 in DCK-LEACH for the vice cluster-head election is as follows.
**Algorithm 2: The vice cluster-head election****Input:** all member nodes and their primary cluster-heads within K clusters **Output:** all vice cluster-heads in the set C2 ----------------------------------------------------------------------------------------------------------------- (1) **For** each cluster (2)     **For** member nodes in the same cluster except the primary cluster-head (3)          Calculate the residual energy objective function $f_{1}(i)$ and the distance function $f_{3}(i)$ of each node, respectively (4)               **If** the sum of residual energy of all nodes is greater than half of the initial energy of the network (0.5 * n$E_{0}$) (5)                 Calculate the node adaptation function value according to Q(i) = 1/2 $*f_{1}(i)$ + ½ * $f_{3}(i)$ (6)               **Else** (7)                   Calculate the node adaptation function value according to Q(i) = $\frac{E_{res}\left(i\right)}{E_{0}}*f_{1}(i)$ + (1 − $\frac{E_{res}\left(i\right)}{E_{0}}$)/2 * $f_{3}(i)$ (8)      Find the node with the largest fitness function value and save this node in formation in the set C2 (9) **End**

An objective function ($f_{3}$) is used for selecting the optimal node that is close to the base station. $f_{3}$ in DCK-LEACH is calculated as shown in Equation (11):

(11) $$f_{3}(i)=\frac{\frac{1}{n}\underset{i=1}{\overset{n}{\sum }}d_{toBS}\left(i\right)}{d_{toBS}\left(i\right)}$$

where, $d_{toBS}\left(i\right)$ is the distance from node i to the base station. 

Equation (11) indicates that when the node is closer to the base station, the larger the value of $f_{3}$, the higher the chance of becoming a vice cluster-head.

For the second factor, we prefer to select the node with the largest possible residual energy among the same cluster. Therefore, the objective function of residual energy in this election is shown in Equation (6) as the same as that of the primary cluster-head selection.

Considering the above two factors, the node with more residual energy and positioned closer to the base station is more likely to become the vice cluster-head. We have the formula as follows:

(12) $$Q(i)=\mu f_{1}(i)+\varphi f_{3}(i)$$

where μ and φ are weighting parameters. At the beginning, we set μ = φ = 1/2. If the sum of residual energy of all nodes is less than half of the sum of the initial energy of the network (0.5 * n$E_{0}$), the update formula of μ and φ are as follows:

(13) $$\mu \left(i\right)=\frac{E_{res}\left(i\right)}{E_{0}}$$

(14) φ(i)=1−μ(i)

where, $E_{0}$ is the initial energy of node i, and $E_{res}\left(i\right)$ indicates the residual energy of node i.

Finally, for each node, the adaptive function value (*Q*) is dynamically recalculated based on the residual energy of the network in the current round, and a node with the largest *Q* is selected as a vice cluster-head within clusters. Finally, K vice cluster-heads are generated.

### 4.3. Stable Operation Phase

When the DCK-LEACH algorithm runs, each node sends its information (location and remaining energy) to the base station in each round, which performs the cluster splitting and cluster-head selection. All nodes communicate with the base station, which consumes a lot of energy. In fact, sometimes it is not useful to re-cluster and change cluster-heads in every round. 

Therefore, in order to reduce this unnecessary energy consumption, we propose a concept to control the frequency of the re-clustering operation, which is similar to the LEACH algorithm in cluster-head selection (r mod (1/*p*)). 

**Figure 2:**
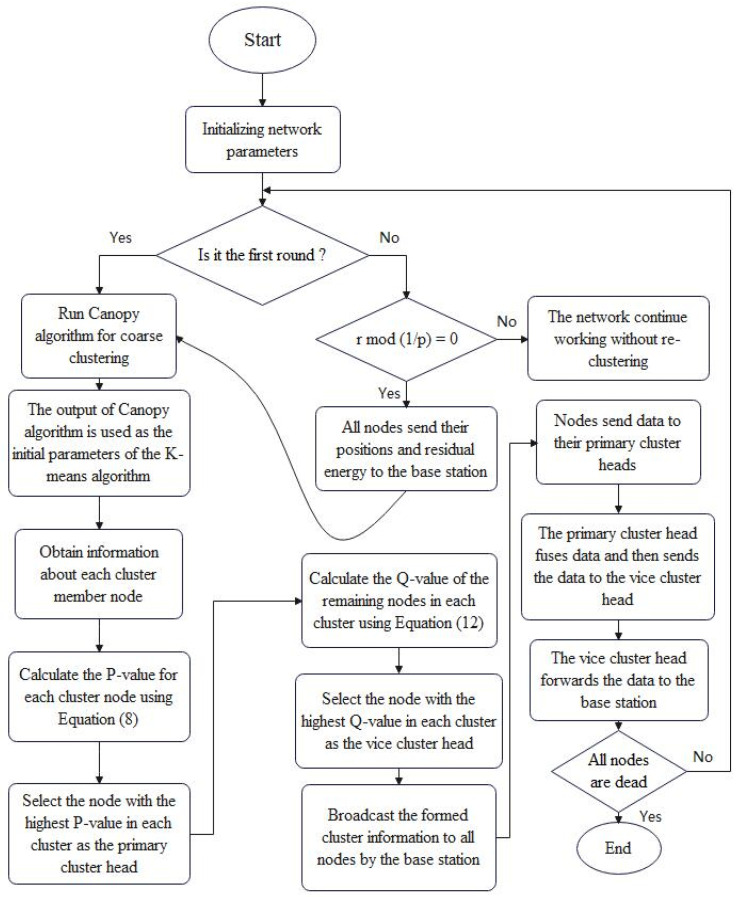
The workflow of proposed protocol.

The clustering and cluster-head selection operations are only performed in a few rounds in an adaptive manner. This is represented here by Cluster, as shown in Equation (15):

(15) $$Cluster=\{\begin{array}{c} 1,\;r=1\\ 1,\;r\;mod\;\left(\frac{1}{p}\right)=0\\ 0,\;Otherwise \end{array}$$

where *r* is the current round, and *p* is a parameter that controls the frequency of the re-clustering operation. *p* value is taken as 0.05 to reduce the communication time with the base station, thus saving energy. When Cluster is equal to 1, it means that re-clustering and cluster-head selection is performed in that round; when Cluster is 0, no clustering operation is performed in the network. Figure 2 illustrates the overall workflow of the proposed algorithm—Algorithm 3 (DCK-LEACH).

After electing the primary and vice cluster-heads from nodes, each node is allocated a cluster based on its position in the network. It sends a message to its primary cluster-head within a cluster. This message includes the node’s residual energy, the distance from its primary cluster-head to itself, and the node’s own ID. 

According to the number of member nodes in the cluster, the primary cluster-head divides the time slots and uses the TDMA (Time Division Multiple Access) mechanism to assign the corresponding time slots to each member node in the cluster [37]. Each node collects and sends data to the primary cluster-head according to the allocated time slots. During non-self-time slots, it remains asleep to save energy.

The primary cluster-head receives the data collected by the member nodes and performs data fusion to delete the redundant data. Then, the fused packets are sent to the vice cluster-head in the cluster, the latter forwards them to the base station. The CSMA MAC (Carrier Sense Multiple Access) [38] mechanism is used to send messages among nodes.
**Algorithm 3:** DCK-LEACH algorithm(1) Initializing network parameters (2) **For** each round (3)     **If** it is the first round (r = 1) (4)        Run Canopy optimized K-means algorithm to get each cluster (5)        **For** each cluster (6)          Select the primary cluster-head (7)          Select the vice cluster-head (8)       Broadcast the formed cluster information to all nodes via the base station (9)       **For** nodes in the network { (10)         Nodes send data to their primary cluster-heads in that cluster (11)         The primary cluster-head fuses data and then sends the data to the vice cluster-head. (12)         The vice cluster-head forwards the data to the base station} (13)    **Else** (14)       **If** r mod (1/*p*) == 0 (15)         All nodes send their positions and residual energy to the base station. (16)         Run again (4) to (12). (17)       **Else** (18)         The network runs without re-clustering (19) All nodes are dead. (20) **End**

### 4.4. Space and Time Complexity Analysis

The time complexity of DCK-LEACH is $O\left(nktf^{2}/c\right)$, where c is the output clustering number of the Canopy algorithm, n is the number of nodes in the network, t is the number of iterations of the algorithm DCK-LEACH, k is the number of clusters generated in DCK-LEACH, and f is the average number of clusters corresponding to each node. In DCK-LEACH, the values of k and c are equal, so the time complexity of DCK-LEACH is $O\left(ntf^{2}\right)$. The space complexity of DCK-LEACH is O(n). 

For the traditional K-means algorithm, the time complexity is O(nkt) and the space complexity is O(n). In DCK-LEACH, the election of both the primary and the vice cluster-heads is determined by three different subordination functions, so the time complexity of the DCK-LEACH is O(n*3) and the space complexity is O(n).

## 5. Results

To verify the effectiveness of DCK-LEACH, we simulate a system where sensor nodes are randomly deployed. We conduct LEACH, K-LEACH, IEECHS-WSN, RCH-LEACH, and DCK-LEACH protocols via Pychar2022.2 software with 16 GB of RAM and Intel Core i5 CPU. Table 2 provides the simulation settings.

The performances of algorithms are evaluated in terms of four aspects: clustering effect, the network lifetime, energy consumption, and throughput. In addition, to verify the performance of the DCK-LEACH algorithm in different dense networks, we performed simulations in three dense scenarios. In addition, the positions of sensor nodes are randomly deployed in the beginning of networks, and multiple experiments are performed to avoid the extreme cases of node’s uneven distribution. 

Three scenarios (1, 2, and 3) are considered (Table 3) in the figures where the notations using parameters are as follows. In three scenarios, the coverage area of the wireless sensor network is in right-angle coordinates (0 ≤ x ≤ 100 m, 0 ≤ y ≤ 100 m), the number of nodes is 100, 200, and 300, respectively, in the network, and the base station is in (50,150) of the network.

### 5.1. Clustering Effect

**Figure 3:**
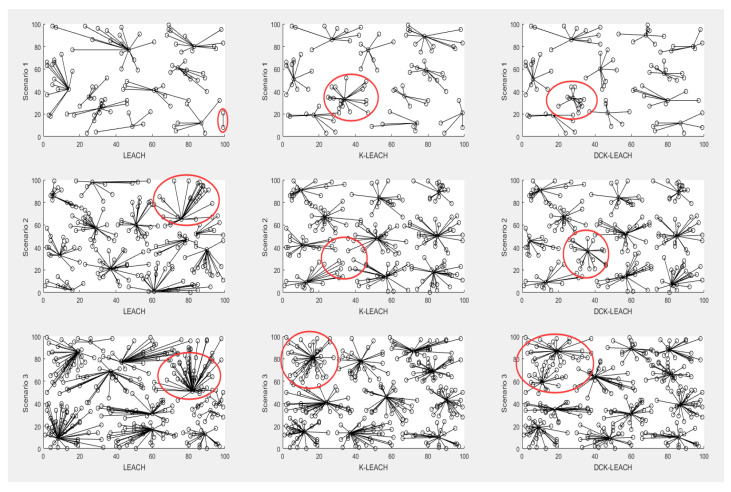
The first round of clustering results in scenario 1, 2, and 3.

The experimental results are shown in scenarios 1, 2, and 3. In each scenario, the results of the first round of clustering of LEACH, K-LEACH, and DCK-LEACH algorithms are shown in Figure 3, which verifies the efficiency of the proposed method. The clustering effects of IEECHS-WSN and RCH-LEACH are similar to LEACH. Due to space limitations, they are not listed here.

In scenario 1 of Figure 3, the LEACH algorithm has the minimal cluster (only 2 nodes) after the first round of clustering, which may lead to uneven energy consumption between clusters. In K-LEACH, the numbers of member nodes in clusters range from 5 to 18, which varies greatly. In DCK-LEACH, the numbers of member nodes in clusters range from 7 to 12, and the maximum number of clusters (12 nodes) is reduced by six compared with that of the K-LEACH algorithm (18 nodes). It shows that the numbers of member nodes in clusters fluctuate little, which means more balance in terms of energy consumption of cluster-heads within clusters.

In K-LEACH of scenario 2, it shows that nodes which are very close to each other (see the nodes within red circles), are not grouped in the same cluster, but join in four rather distant clusters in Figure 3. In DCK-LEACH, the algorithm exactly groups these nodes in the same cluster. This will greatly save energy consumption in data transmission and thus extend the network lifetime.

Scenario 3 of Figure 3 shows that as the number of nodes in the network increases, the LEACH algorithm generated the maximum clusters (49 nodes) and the minimum clusters (7 nodes). Due to the random selection of cluster-heads in LEACH, it is more prone to generating super-scale clusters that significantly shorten the network lifetime. K-LEACH generates the maximum cluster (40 nodes), while the DCK-LEACH algorithm divides these 40 nodes into two clusters, which makes the sizes of clusters more uniform and reduces the energy consumption.

For the results of the LEACH algorithm in all scenarios, since LEACH selects cluster-heads randomly, once the selected cluster-heads are too close to each other, the maximum or the minimum clusters will be generated, which results in the uneven distribution of energy consumption within the clusters. Cluster-heads with a large number of member nodes would be overburdened which leads to premature death.

For the results of the K-means algorithm in all scenarios, because the K-means algorithm is very sensitive to the location of the initial clustering centers, once the choice is unreasonable, a bad effect is had on clustering results. 

Once the nodes are evenly distributed among clusters, their energy consumption within clusters is more uniform, and the network lifetime is significantly extended. 

Meanwhile, DCK-LEACH can solve this problem effectively (see Figure 3), while it uses the Canopy algorithm to calculate and optimize the initial K value of the K-means algorithm, and reduce the possibility of the clustering getting stuck in local optima, the clustering effect is better.

### 5.2. Network Lifetime

The network lifetime is defined as the number of rounds in which the network starts to run until the last node dies.

To better evaluate the network performance, we compare three parameters: FND, HND, and LND of algorithms in three scenarios. The notations used in Table 3 are as follows [39,40,41]:

(1) FND, the number of rounds in which the network starts running until the first node dies;
(2) HND, the number of rounds in which the network starts running until half of the nodes die;
(3) LND, the number of rounds in which all nodes die in the network.

**Figure 4:**
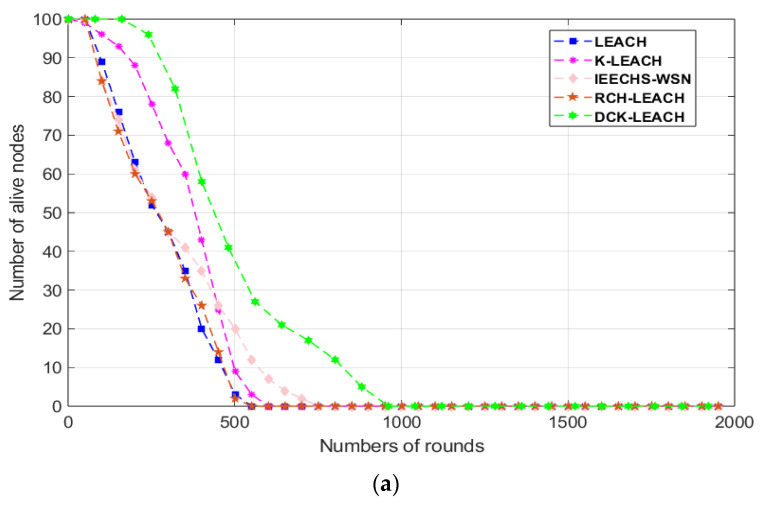

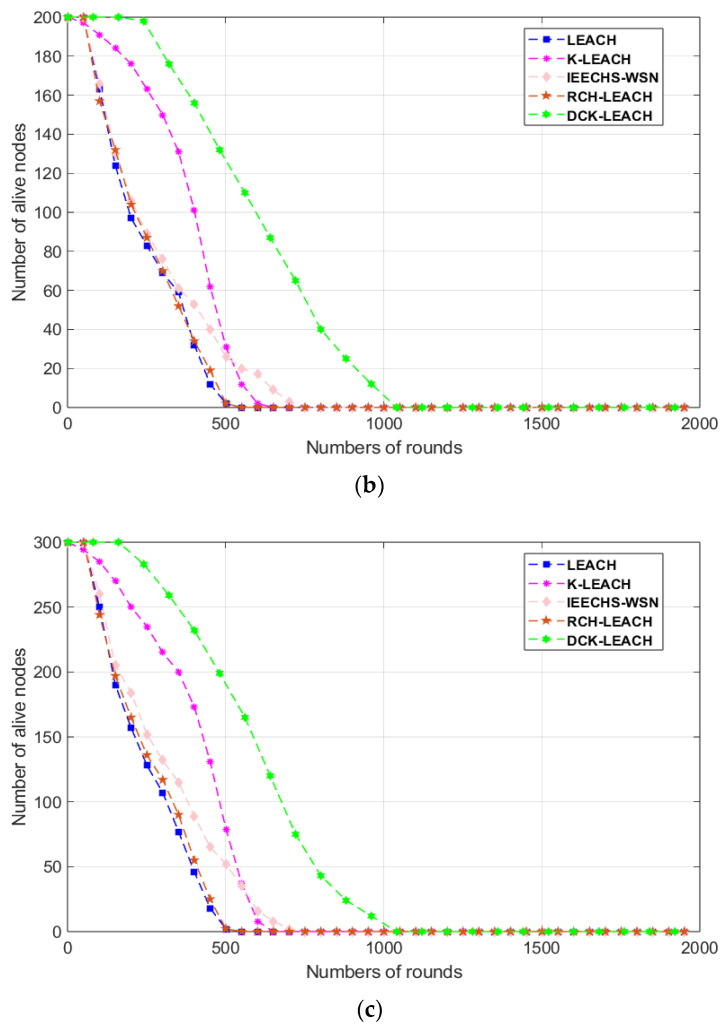
Comparison number of alive nodes in three scenarios: (**a**) 100 nodes, (**b**) 200 nodes, (**c**) 300 nodes.

**Figure 5:**
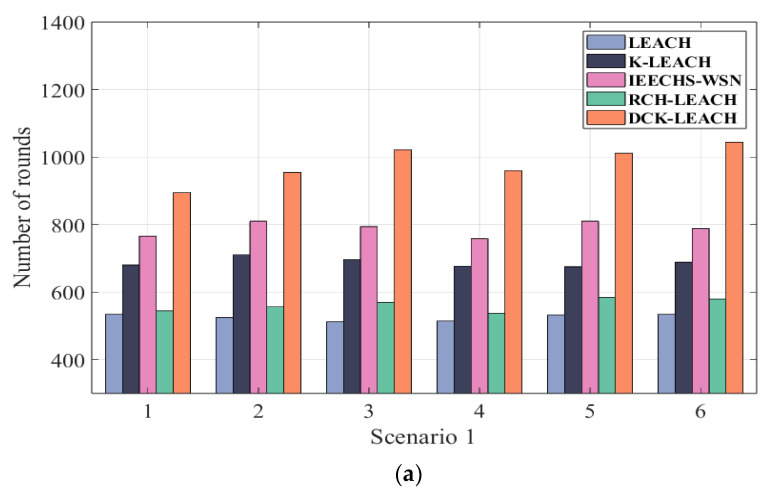

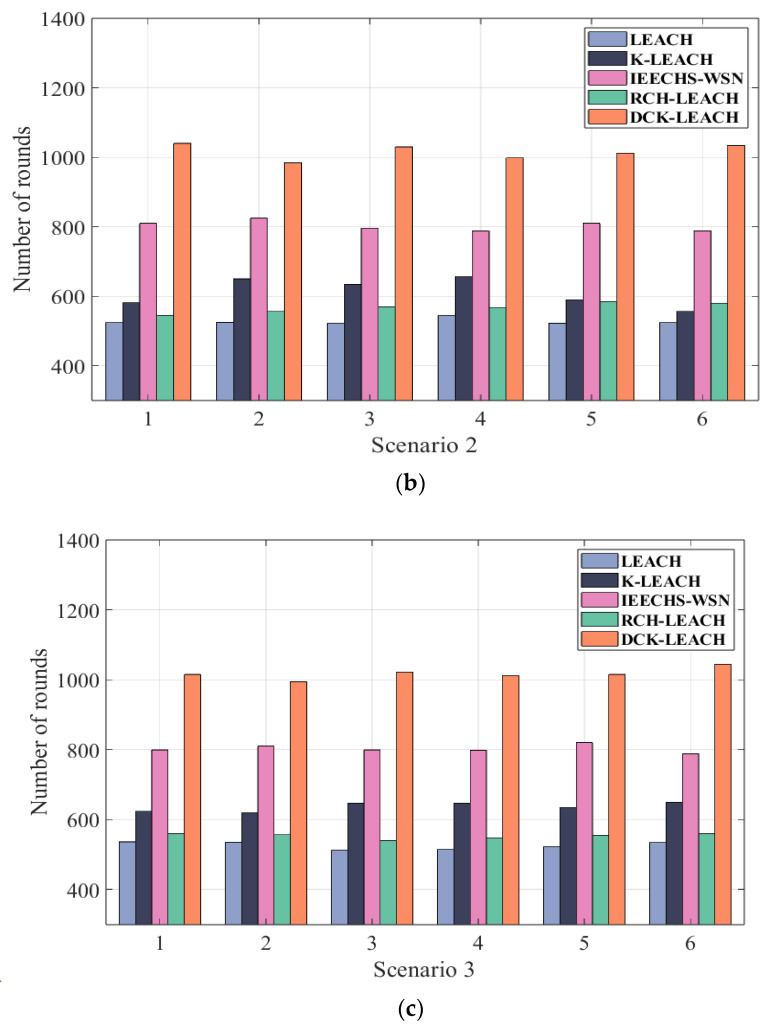
Comparison lifetime of the network in three scenarios: (**a**) 100 nodes, (**b**) 200 nodes, (**c**) 300 nodes. In x-axles of the figure, numbers 1, 2, 3, 4, 5, and 6 are the experiment IDs of multiple executions of the algorithm. Due to space limitations, we only listed the results of six experiments. Other multiple experiment results are similar and are not listed here.

Table 3 shows the results of LEACH, K-LEACH, IEECHS, RCH-LEACH, and DCK-LEACH in terms of FND, HND, and LND in all scenarios. In addition, the simulation results in Figure 4a–c are consistent with that of Table 3. Figure 5a–c show the multiple simulation results of five algorithms in terms of LND (the number of rounds in which all nodes die in the network).

In scenario 1 of Table 3, it is obvious that the FND, HND, and LND of the proposed algorithm are extended compared with that of LEACH (by 55 rounds, 221 rounds, and 534 rounds), K-LEACH (by 43 rounds, 365 rounds, and 680 rounds), IEECHS-WSN (by 62 rounds, 320 rounds, and 766 rounds), RCH-LEACH (by 57 rounds, 225 rounds, and 544 rounds), and DCH-LEACH (by 254 rounds, 485 rounds, and 904 rounds). Compared to LEACH, K-LEACH, IEECHS-WSN, and RCH-LEACH, the improvement of DCH-LEACH in FND is 362%, 490%, 309%, and 345%, in HND that is 119%, 32.8%, 51.5%, and 115%, and in LND that is 69.2%, 32.9%, 18%, and 66.1%. In scenarios 2 and 3, the FND, HND, and LND of our method are significantly longer than that of other protocols. 

In Figure 4a–c, the descending curve in DCK-LEACH is also gentler than that of LEACH, K-LEACH, IEECHS-WSN, and RCH-LEACH. It shows that the network time of DCK-LEACH is the longest compared with that of other protocols.

This is because the DCK-LEACH algorithm adopts the dual cluster-heads mechanism to shorten the communication distance between a single cluster-head and the base station and reduce the energy consumption imbalance caused by the single cluster-head communication mechanism. This reduces the energy consumption of both intra-cluster nodes’ communication and the frequent re-electing of cluster-heads. FND is significantly extended.

Due to reducing the number of re-clustering and communication requests for sending node information to the base station, the inter-cluster energy consumption is effectively reduced; therefore, HND and LND are continuously extended.

Figure 5a–c show the network lifetime of LEACH, K-LEACH, IEECHS-WSN, RCH-LEACH, and DCK-LEACH in all scenarios, where sensor nodes are randomly deployed. In scenario 1, as shown in Figure 5a, in multiple repeated experiments, the network lifetime of DCK-LEACH is between [900 rounds, 1100 rounds], while the network lifetime of LEACH, K-LEACH, IEECHS-WSN, and RCH-LEACH are between [450 rounds, 600 rounds], [580 rounds, 700 rounds], [760 rounds, 850 rounds], and [500 rounds, 600 rounds]. Under the same simulation environment in various repeated experiments, the performance of the proposed algorithm is superior compared to that of other algorithms. 

In scenario 2 and scenario 3, as shown in Figure 5b,c, the same conclusion can be drawn. Other multiple experiment results are similar and are not listed here.

During multiple experiments, the proposed algorithm can effectively prolong the network lifetime in the random deployment environment.

### 5.3. Energy Consumption

While a network is running, the remaining energy of nodes will decrease over time until it becomes zero (i.e., all nodes die) because nodes need to perform various communication tasks and consume energy. The residual network energy could visually reflect the energy consumption of routing protocols. Under the same initial energy of the network, the more residual energy, the lower the energy consumption and the better the performance of the algorithm. 

**Figure 6:**
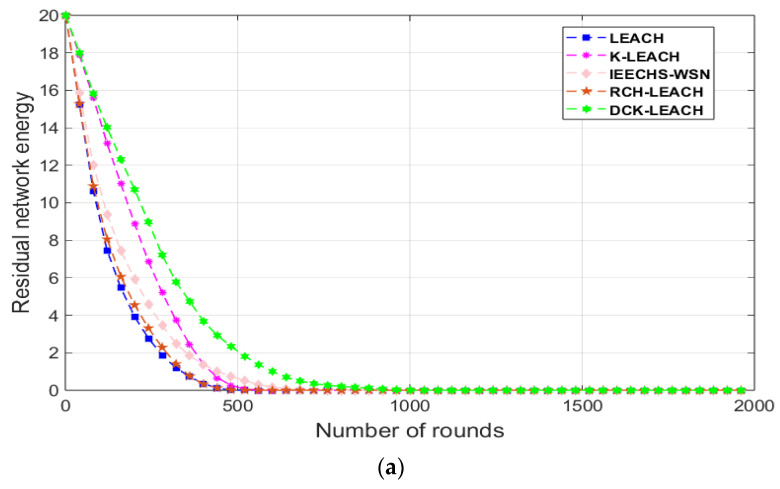

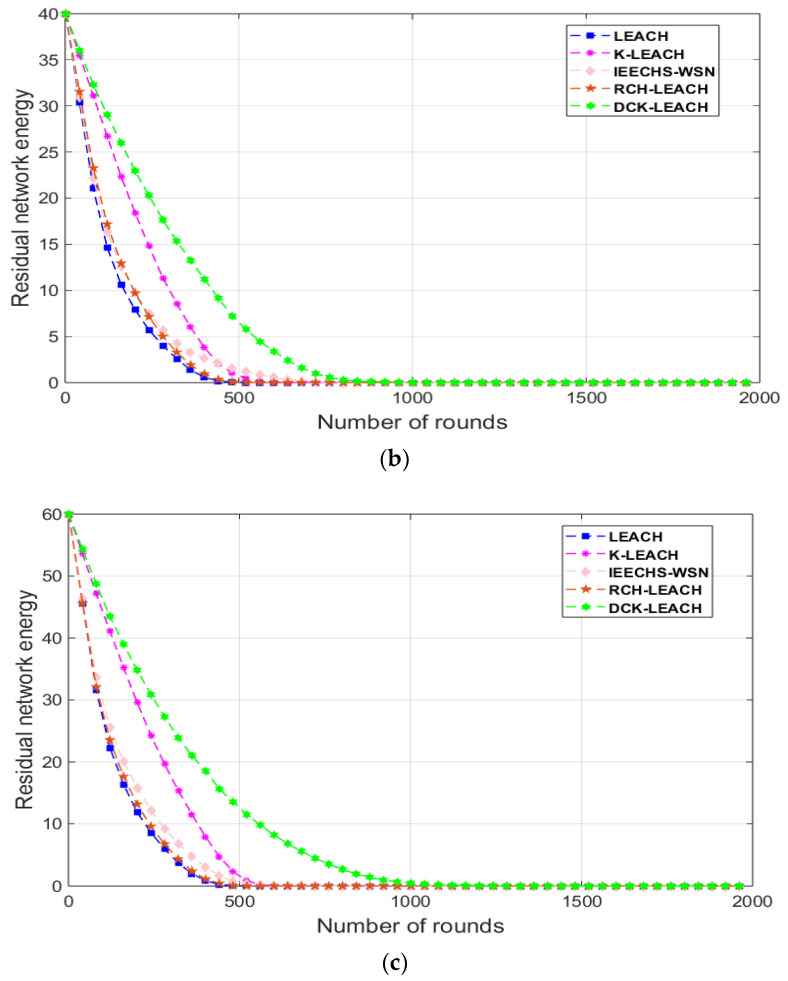
Comparison residual energy of the network in three scenarios: (**a**) 100 nodes, (**b**) 200 nodes, (**c**) 300 nodes.

In scenario 1, as shown in Figure 6a, when the residual energy is half of the initial energy, the rounds of LEACH, IEECHS-WSN, and RCH-LEACH are about 200 because of randomness when clustering. Their performance is not better than the other two algorithms, K-LEACH and DCK-LEACH. K-LEACH carries half of the total energy about 250 rounds, and the DCK-LEACH algorithm is about 300 rounds, which has the minimum energy consumption among the five algorithms.

This is because both K-LEACH and DCK-LEACH algorithms use the K-means algorithm for clustering, so the number of nodes among clusters varies little. In addition, the nodes are evenly distributed among clusters, and as a result, their energy consumption within clusters is more uniform, and the network lifetime is significantly extended. This is shown in Figure 3.

Based on the K-means algorithm, DCK-LEACH optimized the K-value to improve the clustering effect in randomness. Therefore, the DCK-LEACH algorithm is the best in terms of energy consumption. We can obtain the same conclusion from Figure 6b,c in scenarios 2 and 3.

### 5.4. Throughput

Network throughput is an important metric to evaluate routing protocols. Throughput refers to the packets that are eventually transmitted to the base station in the network. In clustering protocols, ordinary nodes send the data they observe to the corresponding cluster-heads. The cluster-head fuses the received data and transmits the packets to the base station after a series of relay. As the network runs, the number of alive nodes in the network decreases, and the packets sent to the base station gradually decrease until the last node dies. The throughput then reaches its maximum value. The higher the throughput, the more effectively information is collected from the surrounding environment, and the better the performance of the protocols.

**Figure 7:**
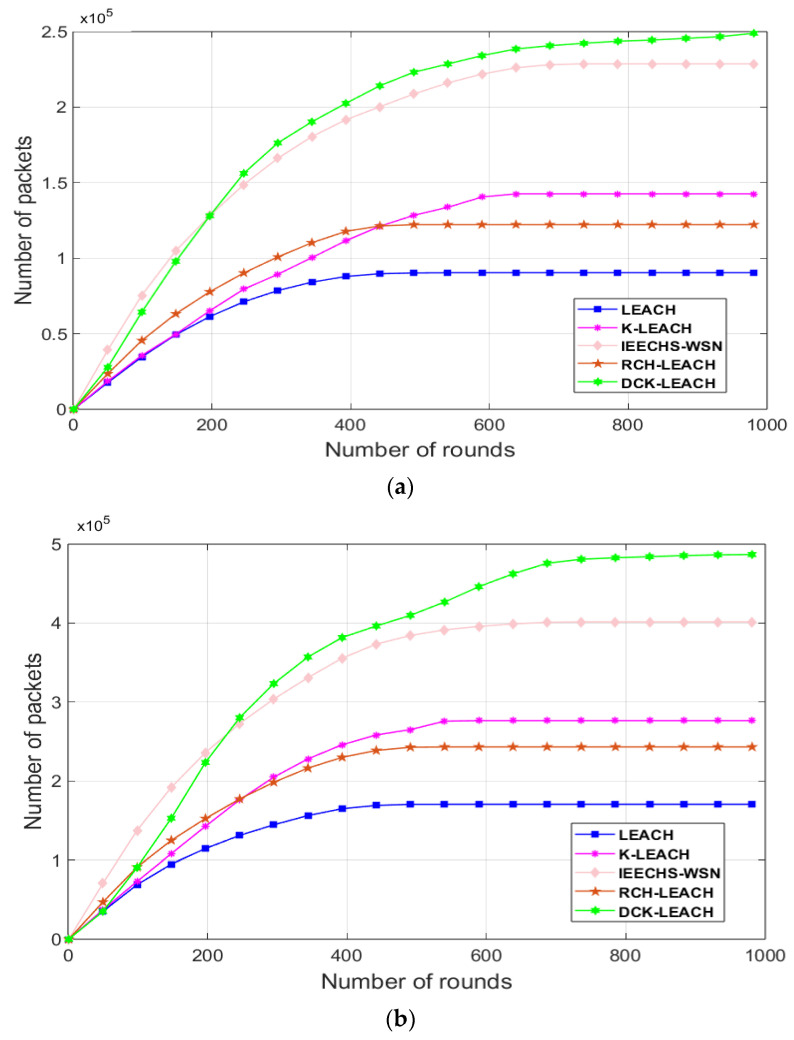

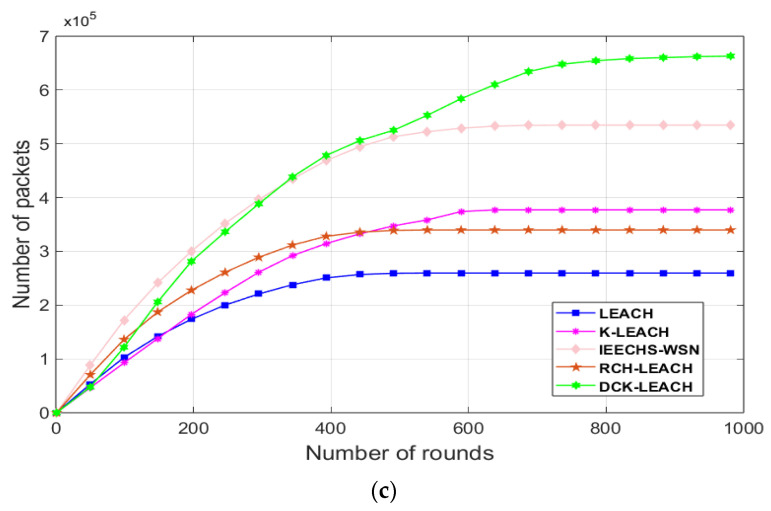
Comparison throughput of the network in three scenarios: (**a**) 100 nodes, (**b**) 200 nodes, (**c**) 300 nodes.

Figure 7a–c show the performance of LEACH, K-LEACH, IEECHS-WSN, RCH-LEACH, and DCK-LEACH in terms of the throughput in all scenarios. In Scenario 1, as shown in Figure 7a, the throughput of DCK-LEACH algorithm is up to 2.5 × 10^5^, and the throughput of the LEACH, K-LEACH, IEECHS-WSN, and RCH-LEACH algorithms are, respectively, 0.9 × 10^5^, 1.4 × 10^5^, 2.3 × 10^5^, and 1.25 × 10^5^. Compared with the other four algorithms, its throughput is improved by 170%, 79%, 8%, and 100%. In the other two scenarios, as shown in Figure 7b,c, DCK-LEACH is superior to LEACH, K-LEACH, IEECHS-WSN, and RCH-LEACH in terms of throughput. This indicates that the higher throughput among them is DCK-LEACH and IEECHS-WSN.

This is because the two algorithms propose the double cluster-heads policies in routing. In addition, the DCK-LEACH algorithm also optimizes in terms of energy evenly distributed, so its network lifetime is longer than that of IEECHS-WSN, therefore, its throughput is slightly higher than that of the IEECHS-WSN algorithm. The cooperation mode of the dual cluster-heads can greatly prolong the survival time of a single cluster-head in a cluster, so sensors can collect more packets and send them to the base station. As a result, its throughput has also been greatly improved.

### 5.5. Scalability in Node Density

In wireless sensor networks, the battery energy of nodes is limited and generally there is no energy supplement. Therefore, the primary goal in designing routing protocols is to let nodes work longer and to efficiently collect and forward more collected data from the source to the base station through the network.

In some practical applications, this will change the network topology through the addition, movement, or failure of nodes from the topology and the failure of the communication link due to environmental factors. At the same time, the increasing of node density will cause the increment of network scale. This requires routing protocols to respond immediately to the change of topology and react to the change of network scale. Therefore, a routing protocol not only needs to have a low energy consumption, but also certain scalability.

In Section 5.2, Section 5.3 and Section 5.4, we design three scenarios in which the number of nodes keeps accumulating. We compare five algorithms based on three aspects in experiments, respectively. As the number of network nodes increases, as shown in Figure 3, LEACH and K-LEACH algorithms generate the maximum clusters. In contrast, the DCK-LEACH algorithm still obtains a relatively good clustering result. 

As shown in Figure 6a–c, with the number of nodes increasing, DCK-LEACH keeps a higher performance in terms of energy dissipation compared with the other algorithms. Therefore, the DCK-LEACH algorithm is salable and can adapt to changes in the node’s density of networks.

### 5.6. Heterogeneous Network Scenarios

For practical applications in wireless sensor networks, not all of the sensors have the same initial energy. In order to compare the performance of DCK-LEACH with the other four algorithms in terms of energy consumption, one heterogeneous network with 300 nodes is considered as Scenario 4, as a 100 m × 100 m network, consisting of two types of nodes:

(a) 20% of all nodes are normal nodes, where the initial energy of nodes is set at 0.1 J;
(b) 80% of all nodes are advanced nodes, where the initial energy of nodes is set at 0.2 J.

**Figure 8:**
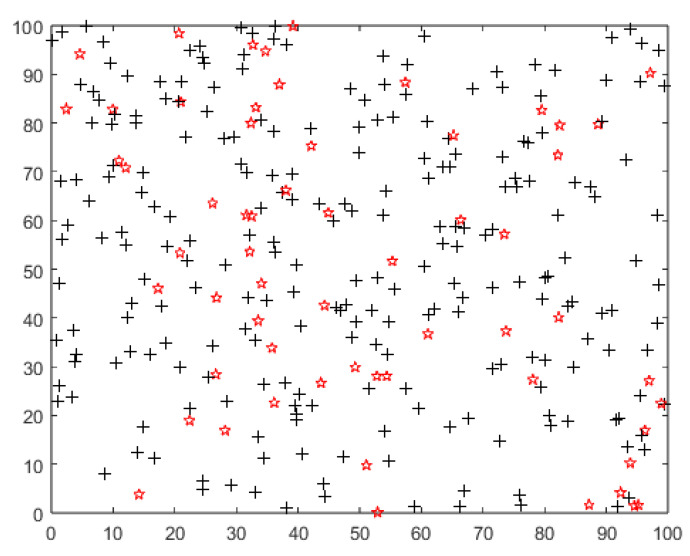
A 100 m × 100 m heterogeneous network with 300 nodes randomly deployed. “+” denotes a node with initial energy of 0.2 J, “red star” denotes a node with initial energy of 0.1 J.

All nodes are randomly deployed, as shown in Figure 8, where the red nodes represent the node (a), and the black nodes represent the node (b).

**Figure 9:**
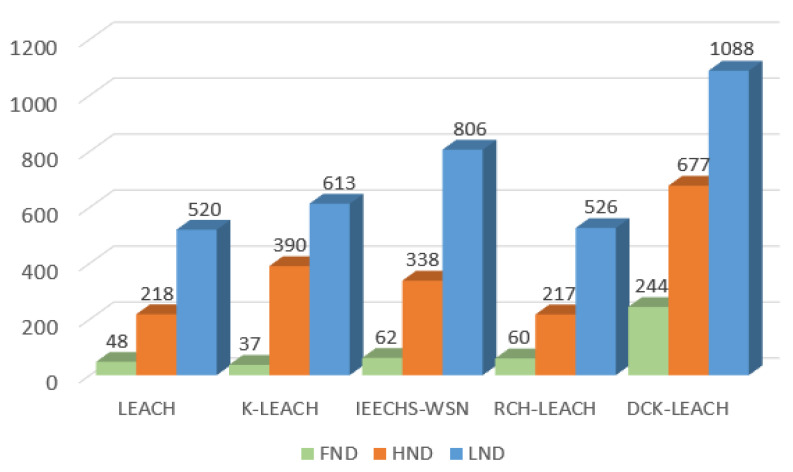
Comparison FND, HND, LND of DCK-LEACH and four other algorithms.

In Figure 9, the FND, HND, and LND of five algorithms in heterogeneous networks are compared. The FND of DCK-LEACH algorithm is 244 rounds, which is 408%, 559%, 293%, and 306% longer than LEACH (48 rounds), K-LEACH (37 rounds), IEECHS-WSN (62 rounds), and RCH-LEACH (60 rounds), respectively. For HND and LND, DCK-LEACH is also the best among the five algorithms.

In Scenario 4, nodes in heterogeneous networks differ in initial energy. Due to frequent re-clustering and different electing of the cluster-head schemes, the performance of other algorithms is degraded to some extent. However, the proposed algorithm does not degrade the performance in terms of FND, HND, and LND in heterogeneous networks, but also improves the performance. It further shows that DCK-LEACH could balance the energy consumption of nodes in the network effectively, and prolong the network lifetime.

**Figure 10:**
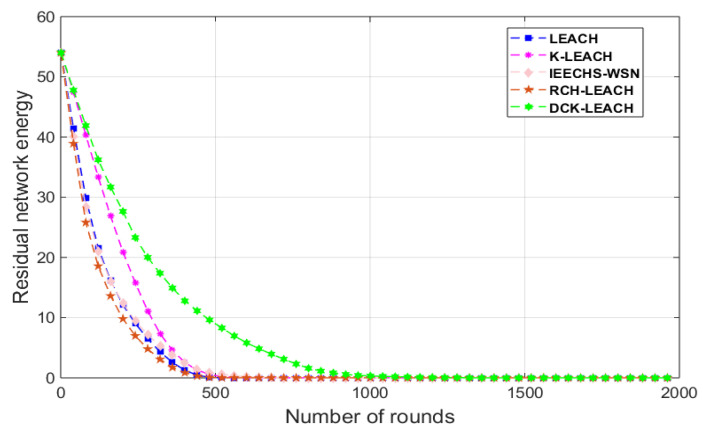
Comparison residual energy of the network.

In Scenario 4, Figure 10 shows the performance of the five algorithms in terms of energy consumption. In Figure 10, compared with that of the other four algorithms, the remaining energy of the DCK-LEACH algorithm decreases the slowest, which indicates that DCK-LEACH could save energy more effectively.

**Figure 11:**
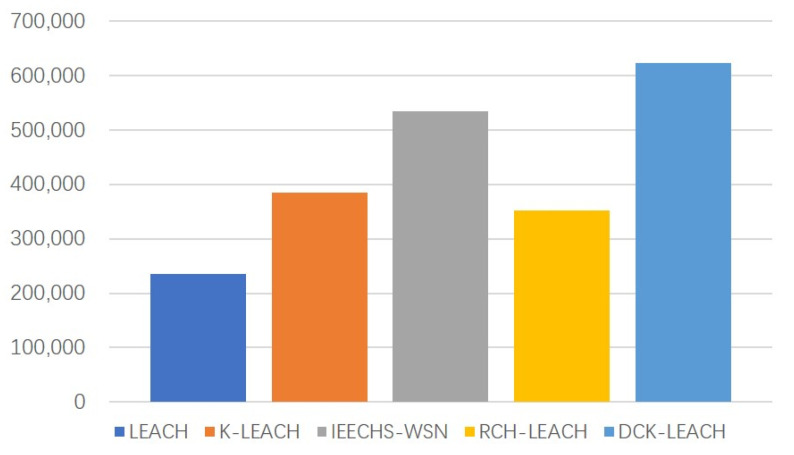
Number of packets (bits) received by the base station during the network is operational.

Figure 11 shows the number of packets received by the base station during the network is operational for the five algorithms. The number of data packets received by the DCK-LEACH algorithm at the base station is significantly higher than that of LEACH, K-LEACH, IEECHS-WSN, and RCH-LEACH. This is because DCK-LEACH abandons LEACH’s random selection of cluster-heads and adopts the double cluster-head mechanism. At the same time, with the change of residual network energy, the energy and distance factors are used to dynamically adjust the weight to select the best double cluster-heads, in order to make energy consumption of the network more uniform. When the network cluster structure is more uniform, the network transmission is stable for a long time. As a result, more data packets are sent to the base station.

## 6. Discussion

This paper tackles an energy-efficient routing problem with a sink in a wireless sensor network. It tackles this problem as two goals by (a) reducing the energy consumption of cluster-heads, and (b) making the energy between clusters more even, i.e., the nodes are evenly distributed in clusters, as a result, their energy consumption within clusters is more uniform.

For the first goal, this division of dual cluster-heads effectively reduces the energy consumption of cluster-heads and improves the lifetime of the network. 

For the second goal, the K-means algorithm is used to cluster sensor nodes. However, the K-means algorithm needs to randomly select multiple nodes as the initial clustering center in the early stage of operation, which may lead to the bad clustering effect. To solve this problem, we introduced the Canopy algorithm, and the nodes calculated by the Canopy algorithm were used as the initial clustering center of the K-means algorithm. This gave a better clustering effect.

In the future, network coding could be conducted as a follow-up to this paper. Network throughput may be improved, and our algorithm could be adjusted to work with delay-sensitive applications.

## 7. Conclusions

This paper proposes a dual cluster-head energy-efficient routing algorithm for WSN. Firstly, the sensor network is divided into multiple clusters. In each cluster, there are three types of nodes: primary cluster-heads, secondary cluster-heads, and common member nodes. Each type of nodes undertakes different tasks in the cluster. Secondly, the common nodes mainly collect all the data and send them to the primary cluster-head, then they fuse the data and send them to the secondary cluster-head, which forwards them to the base station. 

The proposed algorithm dynamically adjusts the weighting factors to select cluster- heads. At the beginning of the network operation, the node’s energy is abundant, therefore, our approach chooses the high energy nodes as cluster-heads, or nodes positioned closer to the centroids of clusters. When half of the nodes in the network are dead, our approach prefers to choose the high residual energy nodes as cluster-heads. The network lifetime can be extended by self-adjustment.

This paper proposes a method to control the frequency of the re-clustering operation and thus avoid wasting energy. Normally, in centralized routing algorithms, all nodes need to send their own information (i.e., location and remaining energy) to the base station, which will waste a lot of energy.

Both in homogeneous and heterogeneous networks, experimental results show that the network lifetime of the DCK-LEACH algorithm is longer compared with that of LEACH, K-LEACH, IEECHS-WSN, and RCH-LEACH, respectively. Therefore, DCK-LEACH effectively reduces the energy consumption of nodes, and significantly prolongs the network lifetime.

## Figures and Tables

**Table 1:** Comparison of function values of member nodes in the cluster.

Node ID	$f_{1}(i)$	$f_{2}(i)$	*p*-Value
1	0.9836	0.8627	0.9132
2	1.0341	0.8168	0.9255
3	0.9807	0.7026	0.8417
4	0.8169	0.9837	0.9003
5	0.7026	1.0341	0.8684
6	1.3911	1.0356	1.2134
7	0.8141	0.9807	0.8974
8	0.7544	1.0400	0.8676
9	1.0504	0.8867	0.9686

**Table 2:** Experimental setting.

Parameter	Value
Simulation Environment	Pychar2022.2
System Properties	Intel Core i5 CPU and 16 GB of RAM
Area	100 m × 100 m
Number of nodes	100, 200, 300
Base station’s coordinates	(50, 150)
Data packet size	4000 bit
Broadcast packet size	100 bit
Node’s initial energy $E_{0}$	0.2 J
$E_{elec}$	50 nJ/bit
$E_{da}$	$50\;\mathrm{nJ}/\left(\mathrm{bit}\cdot {\mathrm{packet}}^{-1}\right)$
$\varepsilon_{fs}$	$10\;\mathrm{nJ}/\left(\mathrm{bit}\cdot m^{-2}\right)$
$\varepsilon_{mp}$	$0.0013\;\mathrm{nJ}/\left(\mathrm{bit}\cdot m^{-4}\right)$

**Table 3:** Comparison FND, HND, LND of LEACH, K-LEACH, IEECHS-WSN, RCH-LEACH, and DCK-LEACH.

Scenario	Number of Nodes	Methods	FND	HND	LND
1	100	LEACH	55	221	534
		K-LEACH	43	365	680
		IEECHS-WSN	62	320	766
		RCH-LEACH	57	225	544
		DCK-LEACH	254	485	904
2	200	LEACH	51	212	524
		K-LEACH	33	398	581
		IEECHS-WSN	61	340	810
		RCH-LEACH	58	227	534
		DCK-LEACH	248	654	1041
3	300	LEACH	56	221	536
		K-LEACH	36	396	623
		IEECHS-WSN	60	322	799
		RCH-LEACH	61	227	538
		DCK-LEACH	238	667	1058

## Data Availability

The study did not report any data.
